# Supplementary material for: Prophage rates in the human microbiome vary by body site and host health
Source: bioRxiv. 2023 May 5:2023.05.04.539508. Preprint. [Version 1] doi: 10.1101/2023.05.04.539508 (PMC10187302; doi:10.1101/2023.05.04.539508)
Supplement: Supplement 1 [file media-1.pdf]

Key resources table

REAGENT or RESOURCE	SOURCE	IDENTIFIER
Deposited data		
NCBI Genome Assemblies Summary Archive 20220601	(1)	https://doi.org/10.25451/flinders.22299664.v2
Prophage predictions	(1)	https://doi.org/10.25451/flinders.c.6629843
Archive of the PATRIC Metadata from 20220601	(1, 2)	https://doi.org/10.25451/flinders.22299655.v2
Prophages in humans	This paper	https://doi.org/10.25451/flinders.22758359.v1

References

1. McKerral JC, Papudeshi B, Inglis LK, Roach MJ, Decewicz P, McNair K, Luque A, Dinsdale EA, Edwards RA. 2023. The Promise and Pitfalls of Prophages. bioRxiv.

2. Wattam AR, Abraham D, Dalay O, Disz TL, Driscoll T, Gabbard JL, Gillespie JJ, Gough R, Hix D, Kenyon R, Machi D, Mao C, Nordberg EK, Olson R, Overbeek R, Pusch GD, Shukla M, Schulman J, Stevens RL, Sullivan DE, Vonstein V, Warren A, Will R, Wilson MJC, Yoo HS, Zhang C, Zhang Y, Sobral BW. 2014. PATRIC, the bacterial bioinformatics database and analysis resource. Nucleic Acids Res 42:D581-91.
